# sPAGM: inferring subpathway activity by integrating gene and miRNA expression-robust functional signature identification for melanoma prognoses

**DOI:** 10.1038/s41598-017-15631-y

**Published:** 2017-11-10

**Authors:** Chun-Long Zhang, Yan-Jun Xu, Hai-Xiu Yang, Ying-Qi Xu, De-Si Shang, Tan Wu, Yun-Peng Zhang, Xia Li

**Affiliations:** College of Bioinformatics Science and Technology, Harbin Medical University, Harbin, 150081 China

## Abstract

MicroRNAs (miRNAs) regulate biological pathways by inhibiting gene expression. However, most current analytical methods fail to consider miRNAs, when inferring functional or pathway activities. In this study, we developed a model called sPAGM to infer subpathway activities by integrating gene and miRNA expressions. In this model, we reconstructed subpathway graphs by embedding miRNA components, and characterized subpathway activity (sPA) scores by simultaneously considering the expression levels of miRNAs and genes. The results showed that the sPA scores could distinguish different samples across tumor types, as well as samples between tumor and normal conditions. Moreover, the sPAGM model displayed more specificities than the entire pathway-based analyses. This model was applied to melanoma tumors to perform a prognosis analysis, which identified a robust 55-subpathway signature. By using The Cancer Genome Atlas and independently verified data sets, the subpathway-based signature significantly predicted the patients’ prognoses, which were independent of clinical variables. In the prognostic performance comparison, the sPAGM model was superior to the gene-only and miRNA-only methods. Finally, we dissected the functional roles and interactions of components within the subpathway signature. Taken together, the sPAGM model provided a framework for inferring subpathway activities and identifying functional signatures for clinical applications.

## Introduction

A comprehensive view necessitates the development of computational strategies for linking gene expression levels to sample phenotypes. The identification of gene signatures has therefore become a major application for many aspects of tumor analyses, including diagnosis^1^, prognosis^2^, recurrence^3^ and response to treatment^4^. However, the gene components of distinct signatures displayed no significant overlap, even though they paradoxically occupied efficient powers in their respective cohorts^5^. One explanation for the lack of gene overlap or low reproducibility of the genetic makeup was that different gene components are merely separate aspects of the same group of biological mechanisms or molecular pathways. Relative to gene signatures, functional signatures that represent sets of gene units with consistent functional roles could display a more robust performance. Moreover, transcriptomic data are usually poorly dimensioned with many more variables than the number of samples, so function-based analyses could reduce the dimensions by incorporating higher-order information^6–9^. It was therefore necessary to infer functional conditions for better interpretation of expression arrays, and to identify mechanistically-derived signatures for the accurate analyses of tumors.

Numerous methods have been recently developed to analyze tumor phenotypes based on their functions or mechanisms. Ooi *et al*. used a computational approach to identify connections between molecular pathways and tumor profiles, and new patterns of driving pathways were identified to define subgroups of gastric cancers, which were clinically relevant to patient survival^10^. Moreover, Huang *et al*. combined Cox proportional hazard regression and L1-lasso penalized features to propose a pathway-based model to predict survival of breast cancer patients^11^. To increase the utility of multi-gene mechanism signatures, a novel method named FAIME was developed to generate “personal mechanism signatures” based on gene expression levels. FAIME computed mechanism scores using rank-weighted gene expressions derived from samples, and was reported to be useful for clinical deployment and available for personal mechanism interpretations^8^. Moreover, many studies have identified and detected individualized dysregulated pathways involved in multiple disease analyses^12–16^. However, most current methods characterized the functional conditions when only considering gene expression levels, and ignored the involvement of regulatory molecules such as non-coding RNAs.

As a major class of non-coding RNAs, microRNAs (miRNAs) regulate gene expression by binding to the 3′-untranslated region (3′-UTR) of messenger RNAs (mRNAs) at the post-transcriptional level. The miRNA-induced inhibition of transcription processes was reported to affect tumor initiation and progression processes^17–19^. Furthermore, the miRNA regulation of genes was involved in pathway activity, and an increasing number of studies have performed pathway-level analyses by including the regulatory roles of miRNAs. Kretschmann *et al*. conducted pathway enrichment analyses by considering miRNA levels^20^. The integration of miRNA and mRNA biomolecules was necessary for interpreting heterogeneous diseases, and has been applied to many disorders, including the identification of breast cancer markers^21^, tumor miRNA signature optimization^22^, and glioma tumor mechanism analyses^23^. In our previous study, a glioma survival network was constructed by simultaneously considering miRNA, gene expression, and pathway topology, and the modules derived from the network were effective in predicting patient clinical outcomes^19^. These findings further confirmed the biological involvement of miRNA molecules; however, the regulatory role of miRNAs has been seldom considered in inferring pathways or functional activities.

Biological pathways have advantages over functional terms owing to their topology structure information. In the meanwhile, the large number of components within total pathways presents difficulties in the analyses. Therefore, the subpathway concept, the pathway region within the whole pathway, was defined based on the pathway topology information from our previous study^24^. Because of the small number of genes, the subpathway reflects more detailed functional descriptions and provides important information necessary to interpret relevant biological phenomena. In our previous study, abnormalities of subpathway conditions were shown to be associated with the etiology of multiple diseases^24,25^. In addition, subpathway-based analysis was used to analyze drug actions, and a drug-subpathway network was constructed for the systematic characterization of drug mechanisms^26^. We have also recently identified prognostic signatures for lung cancer patients based on risk subpathways derived from the cell cycle pathway^27^. Moreover, there also exist other pathway quantification methods which take into account the subpathway level, such as subSPIA^28^, Hipathia^29^, DEAP^30^, and CLiPPER^31^. It can be concluded that the subpathway-based analysis is necessary to infer functional activities for tumor biological interpretations.

We hypothesized that molecular mechanisms obtained from gene and miRNA expression profiles could be used as genome-wide measurements of single sample at the subpathway level. Here, we developed a novel model called the subPathway Activity by integrating Gene and MiRNA (sPAGM) to infer the subpathway functional activity for single samples. The sPAGM model determined subPathway Activity (sPA) scores by using the expression levels of genes and miRNAs within corresponding subpathway graphs. We found that the sPA scores could distinguish different samples from multiple tumor types, as well as between samples from normal and tumor conditions. To illustrate the usefulness of this methodology, we next used sPAGM to identify a mechanism-based prognostic signature for melanoma patients. Melanoma annually causes approximately 50,000 deaths worldwide, and accounts for 0.1% of total global mortality^32^. The clinical management of melanoma is challenging because of the variable survival outcomes. For example, the 5-year survival estimates of melanoma patients with nodal metastatic tumors range from 29% to 81.5%^33^. An accurate prognosis could stratify patients entering clinical trials, and could assist in making decisions regarding the costs and risks of adjuvant treatments. In the present study, a 55-subpathway signature was identified, which was able to predict patients’ clinical outcomes using The Cancer Genome Atlas (TCGA) and verified data sets. We also determined the functional roles and interactions of components within the subpathway signature. In summary, the sPAGM model provided novel insights into characterizations of biological mechanisms at the subpathway level, and provided a framework for detecting and identifying functional signatures that could be used in the prognoses of cancer patients.

## Material and Methods

### Data sets

#### TCGA skin cutaneous melanoma (SKCM) training data set

The SKCM data set was obtained from the TCGA database, and was comprised of gene expressions, miRNA expressions, and clinical data from melanoma patients. The gene expressions were generated using the IlluminaHiSeq_RNASeqV2 platform (Illumina, San Diego, CA, USA), and the miRNA expressions were generated using the IlluminaHiSeq_miRNASeq platform (Illumina). For the expression data, we used the level three data, which provided the expression levels for each miRNA and gene per sample after quantile normalizations and background correlations, and the average miRNA and gene expression values were calculated for duplicated samples. In addition, we excluded samples from patients with survival times <30 days, because these patients might have died for reasons other than the disease^34^. Finally, the expression and clinical data of 325 patients who fit the aforementioned criteria were used as a training set for the identification of prognostic signatures.

#### Melanoma validation data set

The prognostic signatures identified by our model were further validated using an independent melanoma data set obtained from Jayawardana *et al*.^35^. The sample clinical information and expression data included miRNA and gene levels that were obtained from the Gene Expression Omnibus database, and the gene expressions were quantified using an Illumina humanWG-6 beadchip, version 3.0 (Illumina; data accession number: GSE54467) and the miRNA expression was quantified using an Agilent-031181 (Agilent, Santa Clara, CA, USA) unrestricted human miRNA microarray, version 16.0 microarray (data accession number: GSE59334). The sample labels from the miRNA and gene data sets were matched according to the original sample descriptions and clinical characterizations, and the matched miRNA and gene expression for each sample were obtained. Similarly, we eliminated the tumor samples from patients with a survival time < 30 days. 74 melanoma samples were finally included in the validation analyses. When a gene or miRNA had multiple probes, we computed the mean value as the final expression level.

#### Other data sets from TCGA

To test the performance of sPA scores in our model, we utilized the miRNA and gene expression data sets of twelve tumor types for evaluation analyses. These tumor types included bladder urothelial carcinoma (BLCA), breast invasive carcinoma, head and neck squamous cell carcinoma, kidney chromophobe (KICH), kidney renal clear cell carcinoma (KIRC), kidney renal papillary cell carcinoma (KIRP), liver hepatocellular carcinoma, lung adenocarcinoma (LUAD), lung squamous carcinoma (LUSC), prostate adenocarcinoma (PRAD), thyroid cancer (THCA), and uterine corpus endometrioid carcinoma. Using the same previously mentioned procedures, we obtained corresponding miRNA and gene expression data from TCGA level three data, and the average expression values were calculated for duplicated samples. In this analysis, tumor and normal samples of each type of tumor type were analyzed.

### Experimentally verified miRNA–target interactions

We downloaded human specific miRNA–target interactions from the miRTarBase^36^, mir2Disease^37^, miRecords (version 4.0)^38^, and TarBase (version 6.0)^39^ databases. After redundancy processing, 55,146 miRNA–target interactions between 1,110 miRNAs and 20,186 genes were obtained as follows: 50,381 pairs from miRTarBase, 96 pairs from mir2Disease, 518 pairs from miRecords, and 26,388 pairs from TarBase. Among these interactions, a total of 6,459 pairs, involving 358 miRNAs and 3,452 genes, were verified using low throughput experiments^40^.

### Inferring sPAGM

It was necessary to simultaneously consider the expression levels of miRNAs and genes to characterize the functional and pathway activities. Moreover, the subpathways displayed advantages over whole pathway graphs because they provided more detailed information. To combine these data, we proposed a novel statistical model named sPAGM, to infer the sPA score. The input of sPAGM involved a subpathway graph with miRNA and gene components, and an expression matrix with genomic features (including miRNAs and genes) as rows and samples as columns. The objective of sPAGM was to infer sPA scores for each of all biological subpathways based on the expression levels of both gene and miRNA components involved in corresponding subpathways.

#### Reconstruction of subpathway graphs

We reconstructed the subpathway graphs by embedding the miRNA components only if the miRNAs had a regulatory effect on corresponding subpathway genes. The detailed processes involved the following:

i) We first extracted all the biological pathways including 150 metabolic and 150 non-metabolic pathways from the Kyoto Encyclopedia of Genes and Genomes (KEGG) database, and converted them into undirected graphs with gene products as nodes using our previously developed R package^24^.
ii) The *k*-clique method was then used to define subpathways based on the distance similarities among gene products in each pathway. Multiple *k*-cliques in a pathway graph were considered as subpathways where the distance between any two nodes was no larger than *k*, and where different *k* values (2, 3, and 4) were considered and compared in the study.
iii) We next determined whether a miRNA was linked to the *k*-clique subpathway graph based on the verified miRNA–target interactions. The miRNA which regulated at least *t* genes within one subpathway from the low throughput experiments was considered to be embedded into this subpathway graph. The verified interactions between miRNAs and target genes were maintained in the analyses, and different *t* parameters (1–4) were set for comparison.
iv) To reduce bias, we further eliminated the small scale subpathway graphs with less than one miRNA or three genes. The subpathway graphs, which incorporated regulatory miRNA nodes and miRNA–target interaction edges, were finally reconstructed for model analyses.

#### Calculating the sPA scores

Based on the expression levels of miRNAs and genes within reconstructed subpathway graphs, we further calculated activity scores and characterized the functional conditions for these subpathways by using the OrderedList strategy^8^. For the matched miRNA and gene expression matrices, all expressed miRNAs (set ${N}_{mi}$) and genes (set ${N}_{g}$) from the same sample were respectively sorted in an ascending order according to their expression levels, and then exponential decreasing weights ($w$) were assigned to the ordered miRNAs (${w}_{mi,s}$) and genes (${w}_{g,s}$) as follows:

1 $${w}_{mi,s}=({r}_{mi,s})\cdot ({e}^{\frac{{r}_{mi,s}}{|{N}_{mi}|}})$$

2 $${w}_{g,s}=({r}_{g,s})\cdot ({e}^{\frac{{r}_{g,s}}{|{N}_{g}|}})$$

where ${r}_{mi,s}$ and ${r}_{g,s}$ were the expression ranks for each miRNA and gene in the sample $s$, respectively, and $|{N}_{mi}|$ and $|{N}_{g}|$ were the total number for the miRNA and gene components in the corresponding matrix.

For each reconstructed subpathway graph which was a set of miRNA and gene components, there was a component-set and a complement component-set for both miRNA and gene levels. At the miRNA level, component-set $subpat{h}_{mi,i}$ was defined as miRNA components satisfied $miRNAcomponent\in subpat{h}_{mi,i}$ and the complement component-set ${N}_{mi}\backslash \backslash subpat{h}_{mi,i}$ was further defined. At the gene level, component-set $subpat{h}_{g,i}$ and the complement component-set ${N}_{g}\backslash \backslash subpat{h}_{g,i}$ were also defined. Then, we defined the subPathway Activity score ($sPA$ in equations) at the miRNA ($sPAmi$) and gene ($sPAg$) levels as follows:

3 $$sPAm{i}_{subpat{h}_{i},s}=\frac{1}{|subpat{h}_{mi,i}|}\sum _{mi\in subpat{h}_{mi,i}}({w}_{mi,s})-\frac{1}{|{N}_{mi}\backslash \backslash subpat{h}_{mi,i}|}\sum _{mi\in {N}_{mi}\backslash \backslash subpat{h}_{mi,i}}({w}_{mi,s})$$

4 $$sPA{g}_{subpat{h}_{i},s}=\frac{1}{|subpat{h}_{g,i}|}\sum _{g\in subpat{h}_{g,i}}({w}_{g,s})-\frac{1}{|{N}_{g}\backslash \backslash subpat{h}_{g,i}|}\sum _{g\in {N}_{g}\backslash \backslash subpat{h}_{g,i}}({w}_{g,s})$$

For further integrated analyses, a $z$-type statistic was used to define the normalized subPathway Activity score ($sPA\_norm$) as follows:

5 $$sPAmi\_nor{m}_{subpat{h}_{i},s}=\frac{sPAm{i}_{subpat{h}_{i},s}-\overline{sPAm{i}_{subpat{h}_{i}}}}{S(sPAm{i}_{subpat{h}_{i}})}$$

6 $$sPAg\_nor{m}_{subpat{h}_{i},s}=\frac{sPA{g}_{subpat{h}_{i},s}-\overline{sPA{g}_{subpat{h}_{i}}}}{S(sPA{g}_{subpat{h}_{i}})}$$

where $\overline{sP{A}_{subpat{h}_{i}}}$ was the mean value of subpathway $i$ across all samples, and $S(sP{A}_{subpat{h}_{i}})$ represented the standard deviation. When considering the negative regulatory roles of miRNAs (the miRNAs with higher expression levels could reduce the subpathway activities in which these miRNAs were involved), we finally inferred the $sPA$ by integrating the normalized scores from the miRNA and gene levels as follows:

7 $$sP{A}_{subpat{h}_{i},s}=sPAg\_nor{m}_{subpat{h}_{i},s}-sPAmi\_nor{m}_{subpat{h}_{i},s}$$

### Identifying robust prognostic signatures based on sPA scores

After calculating the sPA scores, a subpathway profile with all subpathways as rows and samples as columns was generated. We further performed bootstrap processes to identify the robust prognostic signatures at the subpathway level. First, we selected 80% of the total samples as the training set and the resting 20% were treated as the testing set. For eliminating the bias, we selected the median survival time as the cutoff and kept the same ratio of good and poor survival samples within these two subsets as the original data set. We performed this process 1,000 times to form 1,000 training subsets. Then, we performed Cox univariate analyses based on these 1,000 training subsets, and significant prognostic subpathways were identified from each analytical process (*P* < 0.05). Each significant subpathway was assigned to an identified counting value which reflected how many times the subpathways were significant in 1,000 analyses, and the robust subpathway signatures were finally identified based on the defined cutoffs.

### The hypergeometric enrichment method for prognostic pathway identification

For a comparison of the methods, we also performed a traditional pathway identification based on hypergeometric enrichment analyses. Prognostic genes and miRNAs were first identified based on the training data sets using the Cox univariate method. We then evaluated the enrichment significance for each reconstructed subpathway graph by considering the overlapping extent of miRNAs and genes, and the *P*-value was calculated as follows:

$$P=1-\sum _{x=0}^{r-1}\frac{(\begin{array}{c}t\\ x\end{array})(\begin{array}{c}m-t\\ n-x\end{array})}{(\begin{array}{c}m\\ n\end{array})}$$

where $m$ were the numbers of the human whole genome and miRNAome, of which $t$ genes and miRNAs were involved in the subpathway under investigation, and the number of prognostic genes and miRNAs was $n$, of which $r$ genes and miRNAs were involved in the same subpathway. When many subpathways were considered, a high false discovery rate was likely to result, so we calculated corrected *P*-values for subpathway results using the Benjamini–Hochberg method. The hypergeometric enrichment method also integrated the miRNAs and genes for pathway identification, although it did not consider the negative regulatory roles of miRNAs.

### Individual pathway-based methods

We performed two representative individual pathway-based methods, GSEA and FAIME, for method comparison. Take the subpathway graph as gene set, we respectively calculated the activity scores using these two methods. For GSEA method, we regarded the expression values as difference to rank these genes and calculated the ES scores. For FAIME method, we transformed the gene expression level into FAIME scores for each sample using the available R package^8^. We utilized the gene expression matrix of 12 tumor types and subpathway graphs (gene sets) to obtain the ES profile and FAIME profile.

### Survival analyses of melanoma subpathway signatures

To test the predictive value, we clustered the tumor samples into two risk groups, involving high-risk and low-risk, based on the subpathway profile using the K-means clustering method (K = 2). Kaplan-Meier (K–M) analyses were performed to compare the survival differences of the patients in these two risk groups, and the significance of differences between groups was tested using the log-rank test. We also performed Cox univariate and multivariate analyses to evaluate the contribution of other independent prognostic factors. In all these survival analyses, a value of *P* < 0.05 was considered as significant.

### Clustering and function analyses

Hierarchical clustering analyses were performed using the correlation (uncentered) and complete linkage method in the Cluster3 software, and corresponding clustering results were displayed using Java TreeView imaging software.

We performed function enrichment analyses for gene and miRNA components. For gene level analyses, we first uploaded genes within the subpathway signatures into the Database for Annotation, Visualization and Integration Discovery (DAVID) to obtain significant Gene Ontology (GO) annotations. DAVID is a functional annotation tool used to interrogate gene sets in over 40 annotation categories^41^, and the significant biological process themes were considered in the analyses. For miRNA components involved in the signatures, we also performed function enrichment analyses using the miEAA tool (http://www.ccb.uni-saarland.de/mieaa_tool/).

## Results

### Reconstruction of subpathway graphs by embedding miRNA components

**Figure 1:**
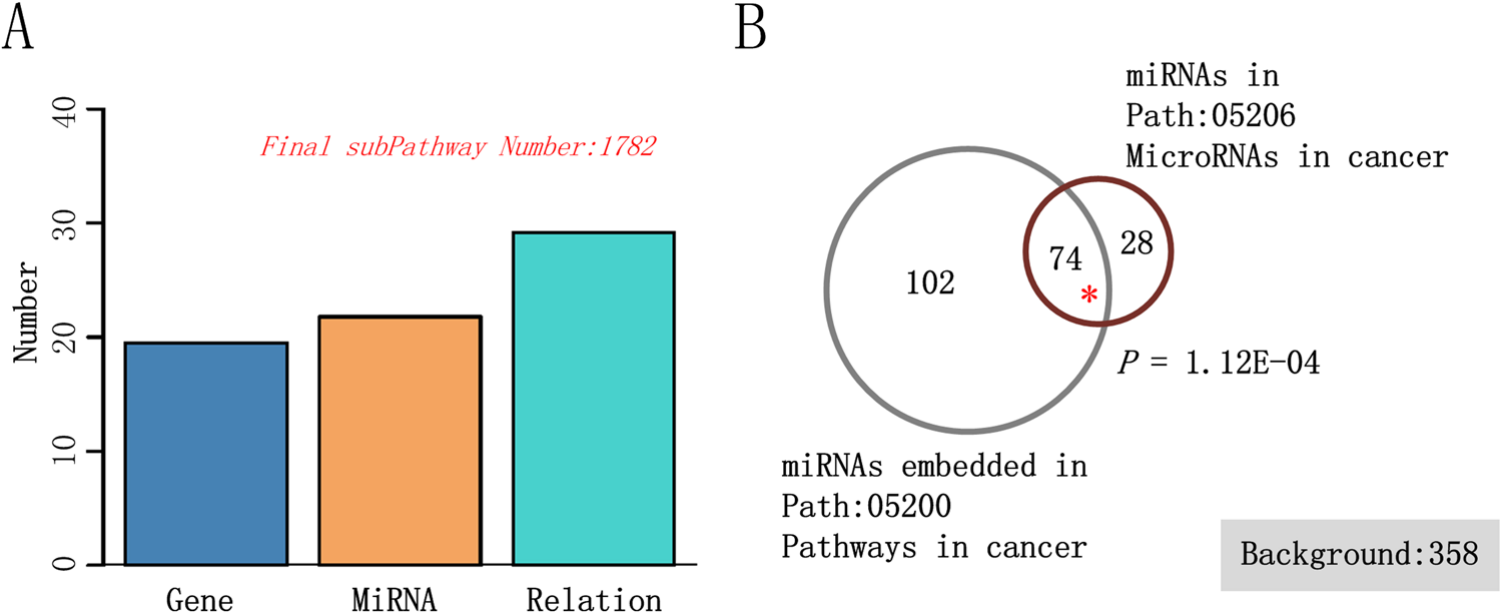
The analyses of reconstructed subpathway graphs. (**A**) The average number of gene nodes, miRNA nodes, and miRNA–gene interactions within each subpathway graph. (**B**) A comparison between the reconstructed pathway graph (Path 05200) and the original pathway graph (Path 05206).

The biological pathways were obtained from the KEGG database, and the miRNA–gene interactions verified by low throughput experiments were downloaded and integrated to reconstruct the subpathway graphs (see Materials and Methods). During this process, different values were assigned to the two parameters, *k* and *t*. The numbers of embedded miRNAs and subpathway scales under different parameter combinations are shown in Supplementary Figure 1. With an increase of *k* values, the components within the subpathway increased; however, the number of all subpathway graphs decreased. The large set of components within the subpathway graph was inconsistent with the subpathway-based analyses. As the number of embedded miRNAs was rapidly reduced with increases of the parameter, *t*, the miRNA–gene integrated analyses became more difficult. After extensive consideration, we finally set the moderate parameters as *k* = 3 and *t* = 1. As a result, a total of 1,782 subpathway graphs were finally obtained, and each subpathway graph contained an average of 21.8 miRNAs and 19.5 genes at the node level, and 29.2 miRNA–gene interactions at the edge level (Fig. 1A).

To test the validity of the reconstructed subpathway graphs, we performed a comparison between components within two specific pathways, Path: 05206 and Path: 05200. The first pathway graph (Path: 05206, MicroRNAs in cancer) was comprised of important miRNAs involved in multiple types of tumors, and the second pathway graph (Path: 05200, Pathways in cancer) was comprised of important gene components involved in tumors. By comparing the miRNA components in Path: 05206 and the embedded miRNAs in reconstructed Path: 05200, we can determine if the embedded miRNAs were functionally involved in the reconstructed pathway graph by calculating the statistical significance. Figure 1B shows that there were 176 miRNAs embedded in Path: 05200, with 74 miRNAs also included in Path: 05206. The hypergeometric test showed that the miRNA overlap was significant (*P* = 1.12E-04) with all 358 miRNAs as background, which confirmed the reliability of the embedded miRNA components in the reconstructed graphs. Moreover, the Path: 05206 graph could be divided into many subpathways according to the tumor type, which were compared with the corresponding cancer pathways (gene components involved) (e.g., Path: 05214 for gliomas). The results for most detailed subpathway cases were also significant as previously mentioned. The comparisons are listed in Supplementary Figure 2.

### The analyses of sPA scores in 12 types of tumors

**Figure 2:**
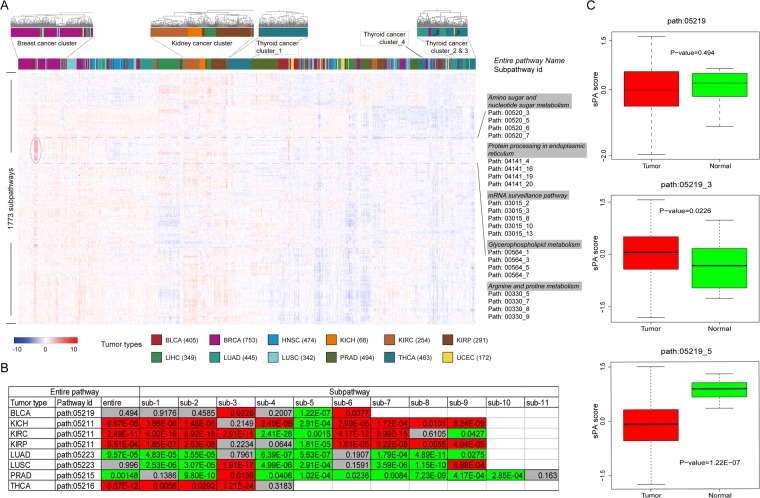
The analyses of subpathway activity scores. (**A**) The clustering results of subpathway profiles with 1,773 subpathways as rows and 4,508 samples as columns. Some sample clusters are shown as examples (above), and different colors correspond to each tumor type. (**B**) The performance of pathway or subpathway activities in distinguishing different conditions. The grey color designates nonsignificant results, The red color designates more activity in tumor samples, and the green color designates more activity in normal samples. (**C**) Path 05219 as an example (the first row in **(B)**.

Based on the reconstructed subpathway graphs, we further inferred the sPA scores for normal or tumor samples by integrating the expression levels of both miRNAs and genes (see Materials and Methods). To test the performance of sPA scores in multiple tumor types, we obtained expression data sets of 12 tumor types from TCGA database, and only the tumor samples were taken into consideration. As described in the Materials and Methods, an activity score was assigned to each subpathway, and an entire subpathway profile with 1,773 subpathways as rows and 4,508 tumor samples as columns was generated. Next, clustering analyses were performed based on the subpathway profile, to determine the differentiation of sPA scores across different tumor types. Figure 2A shows that samples from the same tumor types were clustered together. For example, the breast cancer samples shared consistent sPA scores and formed a close connected cluster, and similar results were also observed in the patients of kidney cancers. These results suggested that there were consistent subpathway activity patterns among patients derived from the same or similar tumor types. In addition, some certain samples of lung cancer and kidney renal papillary cell carcinoma were also included in the breast cancer cluster, which could reflect the potential pathological or physiological similarity among these closely connected samples. For these special samples, the subpathways derived from protein processing, mRNA surveillance, and energy metabolism pathways exhibited high activity.

However, samples from some tumor types were clustered into separate clusters. For thyroid cancer samples, four clusters (clusters _1, _2, _3, and _4) were further formed. It is possible that the samples from these separate clusters displayed different biological processes or characterizations, e,g, patient prognosis. We therefore performed K-M survival analyses to determine whether the clinical outcomes of samples from the above clusters were different. As shown in Supplementary Figure 3, the samples from cluster_3 displayed the best clinical outcomes, while the samples from cluster_2 and cluster_4 displayed the poorest clinical outcomes. Furthermore, the log-rank test showed that there was a marginally significant difference in survival (*P* = 0.074) among the four clusters, indicating the survival relevance of the sPA scores.

### The sPA scores displayed more specificities than the entire pathway-based analyses

Because the results showed the ability of sPA scores to analyze differences and consistencies among tumor types, we determined whether the scores could distinguish tumor and normal conditions. Five tumor pathways were obtained from the KEGG database, and each pathway graph corresponded to one or more tumor types involving Path: 05219 (bladder cancer, BLCA type), Path: 05211 (renal cell carcinoma, KICH, KIRC, and KIRP types), Path: 05223 (non-small cell lung cancer, LUAD and LUSC types), Path: 05215 (prostate cancer, PRAD type), and Path: 05216 (thyroid cancer, THCA type). We then used the sPAGM model to infer the subpathway activities based on corresponding expression data sets, and performed the comparisons between samples from tumor and normal conditions. The Wilcoxon rank sum test was used to test whether these tumor subpathway activities significantly distinguished tumor conditions from the normal state. The activity was also calculated for the entire pathway using our model, and the subpathway-based and pathway-based results were compared. For example, in Path: 05219, one entire pathway and six subpathway graphs were defined and identified, and we inferred the pathway and subpathway activities based on our model. Using TCGA BLCA data set, we also tested the ability of pathway and subpathway activities in distinguishing different tumor and normal conditions. Figure 2B shows that six of eight entire pathways resulted in a significant performance (*P* < 0.05). Four tumor pathways exhibited more activity in tumors and two pathways exhibited more activity in normal conditions. There were two non-significant results for Path: 05219 and Path: 05223, whereas the subpathways from these two entire pathways still displayed significant results. Surprisingly, the different subpathways involved in the same entire pathway displayed different trends, some exhibited more activity in tumor conditions but the others exhibited more activity in normal conditions. Figure 2C shows that the third subpathway of Path: 05219 was more active in tumor conditions (*P* = 0.0226), and the fifth subpathway was more active in normal conditions (*P* = 1.22E-07). These two subpathways were located in separate regions within the entire pathway graph, showing functional independence (Supplementary Figure 4). In a similar manner as BLCA, the differentiation between subpathway and the entire pathway activity were also shown for the other five tumor types, including KICH, KIRC, KIRP, LUSC, and PRAD. Taken together, the results showed that the sPA scores based on subpathway levels displayed more specificities than the entire pathway-based analyses.

### Identification of robust melanoma prognostic subpathways using sPAGM

To determine the possible relevance to clinical applications, the sPAGM model was used to investigate specific tumors such as melanomas. The clinical management of melanoma is challenging, because of variable survival outcomes. The identification of prognostic signatures would facilitate more accurate risk stratification, and would assist in decisions concerning the side effects of adjuvant treatments. Identifying risk subpathway regions could also provide more detailed information for understanding the molecular characteristics of melanomas. TCGA SKCM data sets including miRNA, gene expression, as well as clinical information of corresponding tumor samples, were obtained as training sets to identify prognostic subpathways based on the sPA scores. The detailed process is described in the Materials and Methods.

**Figure 3:**
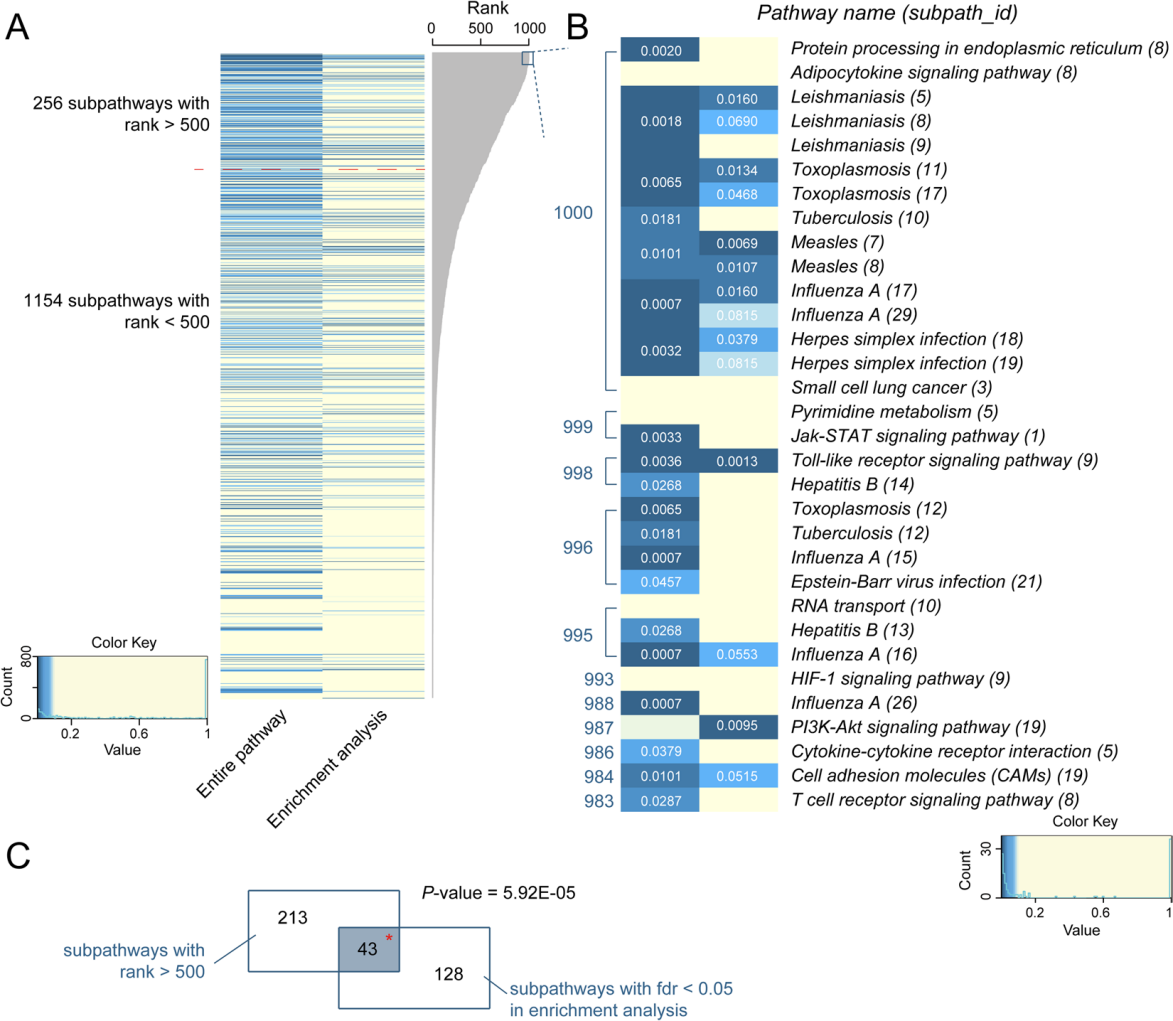
The analyses of melanoma prognostic subpathways. (**A**) Comparative results using sPAGM involving enrichment analyses and the entire analyses. (**B**) Detailed comparative results of subpathways with ranks >980 in **(A)**. (**C**) Overlapping results between our model and the enrichment analysis method.

Based on statistical analyses, each identified prognostic subpathway was assigned to a rank value, which showed the robustness of the corresponding subpathways. For example, a rank value = 500 showed that the subpathway was significantly identified in 500 times of 1000 random analyses. As a result, 256 subpathways had a rank value > 500, and the other 1,154 subpathways had a value < 500. The detailed results of all subpathway ranks are listed in Supplementary Dataset 1. First, we tested whether the entire pathway also displayed significant results for the patients’ survival predictions. Figure 3 shows that significant results were observed in the entire pathway scale from high to low ranks, even if the corresponding subpathway rank was only one. Figure 3B shows that some subpathways from adipocytokine signaling, small cell lung cancer, pyrimidine metabolism, RNA transport, and HIF-1 and PI3K-Akt signaling pathways showed top 30 ranks in subpathway analyses. These results showed that subpathway-based analyses were more informative than the entire pathway analyses. Furthermore, we performed a comparison between our sPAGM model and traditional enrichment analyses for the identification of prognostic subpathways (see Materials and Methods). Overall, 43 of the 256 subpathways with a rank >500 were also significantly identified using the enrichment analyses method (corrected *P*-value < 0.05; Supplementary Dataset 2), with a significance of 5.92E-05 using the hypergeometric test (Fig. 3C). Among the 32 subpathways with rank values >980, 9 subpathways were commonly identified, and some subpathways including pyrimidine metabolism, Jak-STAT signaling, HIF signaling, and the T cell receptor signaling pathways were not significant using the enrichment analyses method. Thus, our sPAGM model identified novel prognostic subpathways, and was a better complementary method than traditional enrichment analyses.

### The advantage of the sPAGM model compared with the miRNA-only and gene-only methods

**Figure 4:**
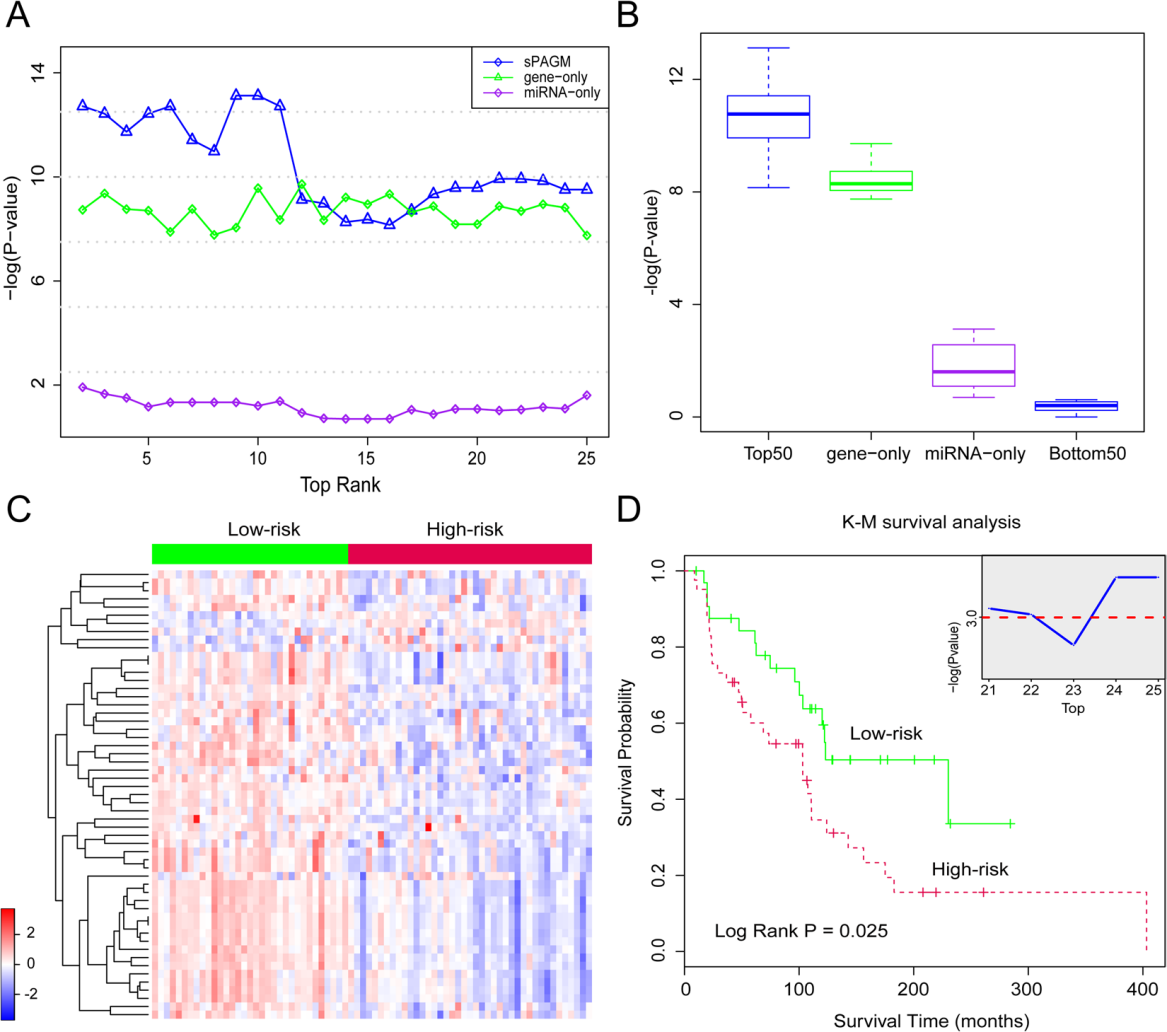
A comparison of methods and survival verification. (**A**) A comparison of the sPAGM model, and gene-only and miRNA-only methods using the top 25 subpathways. (**B**) A further comparison of methods when increasing the top rank value. (**C**) A K-mean clustering representation of the subpathway signatures in the independent data set. The columns include 74 tumor samples and the rows include 55 subpathways. The red colors designate high subpathway activity, and the green color designates low activity. (**D**) A K-M plot of the low risk and high risk groups. The *P*-value was calculated using the log-rank test. Other subpathway signatures with ranks <25 were also tested.

Although the sPAGM model integrated miRNA and gene expression levels to infer the subpathway activity, whether this model was superior to the component-only genomic method needed to be further investigated. We therefore developed two new genomic methods, the gene-only and miRNA-only methods, which respectively considered the gene and miRNA components. Using the TCGA data, we utilized the gene-only and miRNA-only methods to calculate the sPA scores for significant subpathways. Based on new sPA scores, the TCGA samples were divided into high-risk and low-risk groups by K-mean clustering, and P-value was calculated in a similar manner. The significant subpathways were sorted by rank values, and the survival performances of these subpathways with the same ranks calculated using sPAGM, the gene-only, and the miRNA-only methods were compared. Figure 4A shows that sPAGM was superior to the gene-only and miRNA-only methods for survival predictions when the subpathway ranks were >25. When expanding the considered rank value, we also observed that our model was significantly better than the gene-only method (*P* = 2.61E-09), while the miRNA-only method still showed the poorest performance (Fig. 4B). We also performed negative analyses based on subpathways with a bottom rank of 50 to test its reliability. As expected, subpathways with a bottom rank displayed poor predictive performance, which was even worse than the miRNA-only method. Taken together, these results showed the advantage of the sPAGM model over the miRNA-only and gene-only methods for clinical outcome applications. Finally, we defined a total of 55 subpathways with a top 25 ranking as signatures for melanoma subpathway prognoses.

### The subpathways identified by sPAGM predicted melanoma patient clinical outcomes in TCGA and verified data sets

To test the survival predictive power of the 55 subpathway signatures, we performed K-mean clustering (K = 2) to identify the subpathway profile with these subpathways. Two risk clusters, low risk and high risk, were formed, and K-M survival analyses were used to evaluate the signature’s predictions. The results showed that 55 subpathways were significantly associated with the survival status of melanoma patients in TCGA data set (*P* = 7.4E-05; Supplementary Figure 5). To avoid overfitting to a single study, further verification was performed using a completely independent data set from Jayawardana *et al*.^35^. Figure 4C shows that the high risk and low risk groups were also formed for 74 melanoma patients using the verified data set. Consistent with the TCGA results, patients in the low risk group exhibited high subpathway activities, and patients in the high risk group exhibited low subpathway activities. The mean survival time of the low risk group was 112.3 months and the mean survival time of the high risk group was 91.8 months. The log-rank test also showed that there was a difference in survival times between the low risk and high risk groups (*P* = 0.025). When considering subpathways with ranks <25, the survival predictive abilities of these adjacency signatures were also found in the top-24, top-22, and top-21 subpathways (Fig. 4D), further confirming the prognostic robustness of the subpathway signatures.

To test the robustness of survival results, we further performed the permutation and calculated the adjusted P-values for subpathway signatures. First, we permutated the survival time of samples 1,000 times and formed random clinical datas. Based on each of these 1,000 random datas, we calculated the survival P-value for certain subpathway signatures. Finally, the empirical P-value was calculated by counting how many times the random survival P-values were below the real one in 1,000 times. The results showed that our subpathway signatures also displayed the predictive power after random analyses in TCGA data set (adjusted P-value = 0) and verified data set (adjusted P-value = 0.03).

### The subpathway signature predicted melanoma patients’ clinical outcomes independently of clinical variables

Because the predictive power of the subpathway signature was confirmed using multiple data sets, we further performed univariate and multivariate analyses to test whether the signature predicted survival independently of other prognostic factors. Using univariate analyses of TCGA data set, some clinical factors, including age and T stage, were significantly associated with patient survival. Our subpathway signature showed the most significant associations (*P* < 0.0001), and the Hazard Ratio (HR) value showed that it was a risk factor consistent with the K-mean clustering results (Supplementary Figure 5). In the independent data set, all clinical factors were not associated with survival, but our subpathway signature showed significant results (*P* = 0.0429; HR = 1.909). Using multivariable analyses, our subpathway signature predicted the survival when considering other clinical factors in TCGA data set (*P* < 0.0001) and the independent data set (*P* = 0.0342). In conclusion, the subpathway signature predicted melanoma patient survival independently of clinical factors, including age, sex, and stage of the disorder. The detailed results of univariate and multivariate analyses are listed in Table 1.

**Table 1:** Univariable and multivariable analyses of clinical factors and our subpathway signature.

		**Univariable analysis**		**Multivariable analysis**	
		**HR (95% CI)**	**P-value**	**HR (95% CI)**	**P-value**
**TCGA**					
Age	>60/≤60	1.706 (1.195 to 2.437)	0.0033	1.546 (1.065 to 2.244)	0.0219
Gender	Female/male	1.018 (0.701 to 1.478)	0.9262	0.990 (0.679 to 1.445)	0.9603
T	T3, T4/T0, T1, T2	1.814 (1.273 to 2.583)	0.0010	1.465 (1.007 to 2.130)	0.0458
N	N2, N3/N0, N1	1.460 (0.956 to 2.231)	0.0799	1.299 (0.764 to 2.209)	0.3347
M	M1/M0	1.269 (0.402 to 4.013)	0.6845	0.776 (0.239 to 2.520)	0.6732
Stage	III, IV/I, II	1.638 (1.143 to 2.348)	0.0072	1.690 (1.075 to 2.657)	0.0231
Our sig	High risk/low risk	2.212 (1.538 to 3.182)	<0.0001	2.231 (1.531 to 3.252)	<0.0001
**GSE31210**					
Age	>60/≤60	1.585 (0.874 to 2.874)	0.1296	1.825 (0.990 to 3.363)	0.0539
Gender	Female/male	0.877 (0.473 to 1.625)	0.6755	0.833 (0.442 to 1.567)	0.5700
Stage	III/I, II	1.878 (0.879 to 4.011)	0.1036	2.052 (0.935 to 4.503)	0.0729
Our sig	High risk/low risk	1.909 (1.021 to 3.570)	0.0429	1.981 (1.052 to 3.728)	0.0342

### Dissecting the function and interaction of components within the subpathway signature

To further dissect the functional roles of the subpathway signature, we first extracted the gene components and performed DAVID function analyses (see Materials and Methods). As shown in Supplementary Dataset 3, some immune-related terms, including immune response (GO: 0006955) and response to virus (GO: 0009615), were significantly enriched in the gene components of the subpathway signature. Moreover, regulation of some cell processes involving regulation and signaling cascade terms were also enriched. For the miRNA components, we performed function analyses by freely using the tool, miEAA. The results showed that the melanoma disease pathway was the most significantly identified (Supplementary Dataset 4), which showed that the miRNA components played vital roles in melanoma. In addition, DNA damage, cell cycle regulation, and p53 and ErbB signaling pathways were also identified, showing the potential involvement of these processes in melanoma formation and progression.

**Figure 5:**
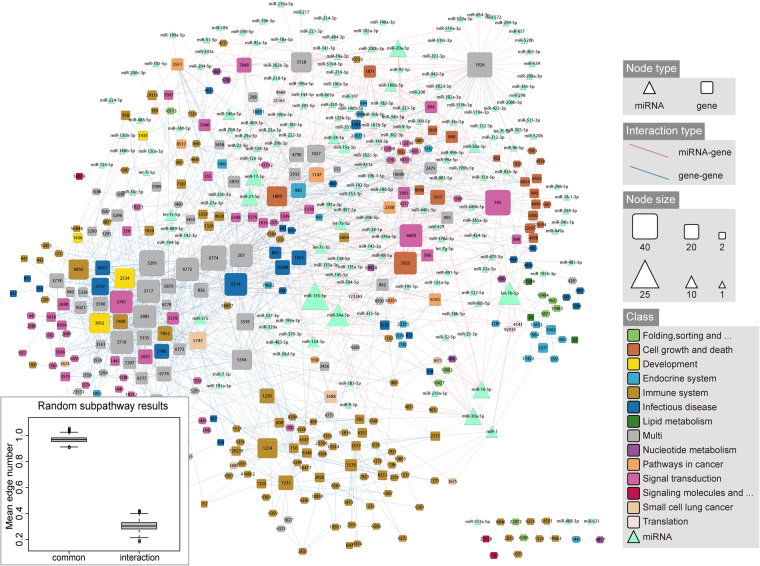
The miRNA–gene interaction network. The triangles and rectangles in the interaction network designate miRNAs and genes, respectively. miRNA and gene node sizes were proportional to the degree of the nodes. Gene nodes were colored according to their pathway categories, which were derived from the KEGG database. The random subpathway results are shown in the lower left quarter.

We then mapped the miRNA and gene components within the subpathway signature into the protein-protein interaction network from HPRD (http://www.hprd.org/). In addition, the miRNA–gene associations were also added to form a miRNA–gene interaction network. Figure 5 shows that the gene and miRNA components were connected into a global network, which were mediated by the miRNA–gene and gene–gene interactions. Moreover, the average distance among the components was 3.46, which was significantly shorter than the random network (4.22 ± 0.06). The gene components belonging to different pathway categories also closely interacted, reflecting the pathway crosstalk within our prognostic signatures. At the subpathway level, 55 subpathways within the signature shared an average of 5.57 genes and miRNA components, and 7.28 gene interactions were derived from the HPRD network between 1,485 subpathway pairs. Figure 5 shows that the results were significantly larger than the random results. There were only 13 subpathway pairs without a gene-interaction relationship. Instead, 6 subpathway pairs among these pairs shared common components. Together, the results showed that the combination of multiple subpathways, rather than individual subpathways, resulted in the best predictive performance.

## Discussion

Although the functional inference of tumor biological mechanisms is already explored, a computational method both considering the miRNA’s regulatory roles and the subpathway scale is urgently needed. In the present study, we developed a novel model, sPAGM, to infer the sPA scores by considering the expression levels of miRNAs and genes. First, we validated the distinguishing performance of the sPA scores among samples across 12 tumor types, as well as the samples between tumor and normal conditions. Comparative analyses showed that sPAGM outperformed the entire pathway-based method. We applied this model to melanoma tumors to identify biological mechanisms at the subpathway level, and to identify prognostic subpathway signatures. By verifying the predictive performance using TCGA and independent data sets, we showed that the subpathway signature displayed a robust predictive power in patient clinical outcomes. Univariate and multivariate analyses showed that the prognostic signature was independent of other clinical factors. Finally, we performed functional and network analyses for components within the subpathway signature to identify functional roles and interactions.

The incidence of melanoma tumors has been increasing in recent decades, and novel methods are urgently needed to identify robust signatures for prognoses. Many gene expression signatures have been identified for this purpose. Winnepeninnckx *et al*. identified 254 genes that were associated with distant metastasis-free survival (DMFS) of melanoma patients. Twenty-three of these genes, which were overexpressed in patients free of metastasis, were thought to be prognostic signatures^42^. In 2013, Brunner *et al*. identified a nine gene signature for melanoma, which was related with the overall survival and DMFS survival^43^. To our surprise, no gene overlap was noted between these two signatures, emphasizing the advantage of functional signatures with more biological relevance and robust performance. We therefore developed the sPAGM model to infer the functional scores at the subpathway level, which was used to identify signatures for the prognosis of melanoma. As a comparison, the gene components within our subpathway signatures shared two genes (CDC6 and IL6) with the Winnepeninnckx’s twenty-three genes.

Even though some approaches have been developed for characterizing functional conditions^8,10,11^, the miRNA component was usually not considered. As regulatory molecules, miRNAs exhibit negative regulatory roles on their target genes at the post-transcriptional level, further affecting protein expression and cellular functions. The functional activity was therefore affected by both the gene expression levels and the regulatory roles of miRNAs. Using our sPAGM model, the subpathway activity was inferred by integrating the expression levels of miRNAs and genes. To test the reliability of the miRNA–gene interactions, we first performed analyses based on two specific pathways involving miRNAs in cancer (Path: 05206) and pathways in cancer (Path: 05200). The comparative results showed that the embedded miRNAs in reconstructed pathway graphs were involved in the corresponding tumor pathways. To compare the methods, we also performed similar procedures based only on gene or miRNA expression, which we named gene-only and miRNA-only models. By testing the survival performance of these models, we observed that our sPAGM model outperformed the miRNA-only and gene-only methods, further showing the necessity of integrating the miRNA and gene components.

To test the performance of our model, we further performed two representative individual pathway-based methods, GSEA and FAIME, for comparison. First, we respectively calculated the subpathway scores (ES scores and FAIME scores) using these two methods (see Materials and Methods). Then, the Pearson correlation between sPA scores and ES scores (or FAIME scores) was respectively calculated. In this process, the common subpathways for individual sample were utilized. As shown in Supplementary Figure 6, our method displayed positive correlation with FAIME method. A possible explanation for the correlation was that similar OrderedList strategies were involved in these two methods. For the GSEA results, no correlation was observed and further comparisons within each tumor type were performed. For most of these tumor types, our model also displayed positive correlations with both FAIME and GSEA methods, especially for the BRCA, KICH and LIHC types (see Supplementary Figure 7). It can be concluded that our sPAGM method displayed consistent activity trend with FAIME method, and acted as a complementary method for GSEA.

The subpathway concept provided detailed gene interaction information and displayed advantages over the entire pathway. Many methods have been developed to perform the pathway analysis based on the subpathway level^28–31^. In our previous studies, the subpathway regions were also defined and identified to characterize the involvement of pathway deregulation in many biological phenomena, including disease occurrence, drug action, and miRNA regulation^26,44,45^. In the present study, we compared the subpathway-based and entire pathway-based methods. Figure 2B shows that the subpathway method was more informative than the entire pathway method, and was able to distinguish tumor and normal conditions. Different subpathways derived from the same pathway resulted in opposite trends in tumor and normal conditions; the sub_5 from the path 05219 displayed high activity in the normal samples, whereas sub_3 and sub_6 from the same pathway displayed high activity in tumor samples. The subpathway method therefore contained more detailed functional descriptions, which could be applied for more accurate prognoses. However, the smaller set of components within the subpathway graph was also unacceptable because of a lack of information. Thus, we defined the subpathways after setting the *k* value as 3 (in the *k*-clique method), and the subpathways with less than one miRNA and three genes were removed.

As a practical application, we used the sPAGM model on melanoma tumors to identify robust survival-related subpathways, with top ranks being identified as prognostic signatures. Survival analyses using TCGA and verified data sets resulted in a 55-subpathway signature, which displayed a robust predictive performance. Among these subpathways, some signaling pathways, including Toll-like receptor signaling pathway, Chemokine signaling pathway, and PI3K-Akt signaling pathway, were all close related with the tumor biology^46–48^. To our surprise, large number of pathways related to infectious diseases. Especially, three subpathways from leishmaniasis were the most robust subpathways. And it was reported that the cutaneous leishmaniasis in hot areas pave the way to the mutation and development of skin cancer^49^. Also we confirmed the signature predicted patient clinical outcomes independently of clinical variables, which especially included the tumor stage. Furthermore, we performed survival analyses to test whether the subpathway signature predicted the clinical outcomes of patients with different stages. Using TCGA data set that contained an adequate number of tumor samples, we found that the signature was also related with the patient’s clinical outcome from early stage to late stage (the number of stage I and II: 132, *P* = 0.94E-03; the number of stage III: 135, *P* = 0.20E-03).

In the present study, we describe a novel integrated sPAGM model based on subpathway graphs and sample-matched miRNA and gene transcriptome. The sample-matched miRNA and gene data sets simultaneously detected the miRNA and gene expression levels for each sample, and provided a more reliable resource to perform miRNA–gene integrated analyses to infer the functional activities. A novelty of our computational model is deducting expressional activity scores of miRNAs from the scores of genes. The direct inhibition of miRNAs to target genes but not other systematic feedbacks or indirect consequences was considered in the calculation of sPA score. Based on this model, we identified risk subpathways as melanoma prognostic signatures to classify tumor samples into different survival groups, and we verified analyses results using another independent data set. Additional performance verification based on sample-matched miRNA and gene data sets could further increase the power of our subpathway signatures for melanoma prognoses. Moreover, the activity score calculated by sPAGM could also be applied to other analyses, including tumor progression and drug action mechanisms for other types of tumors.

## Electronic supplementary material

Supplementary Figures

Dataset 1

Dataset 2

Dataset 3

Dataset 4
