# Supplementary material for: sPAGM: inferring subpathway activity by integrating gene and miRNA expression-robust functional signature identification for melanoma prognoses
Source: Sci Rep. 2017 Nov 10;7:15322. doi: 10.1038/s41598-017-15631-y (PMC5681640; doi:10.1038/s41598-017-15631-y)
Supplement: Supplementary file 1 — Supplementary Figures [file 41598_2017_15631_MOESM1_ESM.doc]

**sPAGM: inferring subpathway activity by integrating gene and miRNA expression — robust functional signatures identification for melanoma prognoses**

***Scientific Reports***

Chun-Long Zhang^1^, Yan-Jun Xu^1^, Hai-Xiu Yang, Ying-Qi Xu, De-Si Shang, Tan Wu, Yun-Peng Zhang^*^, and Xia Li^*^

College of Bioinformatics Science and Technology, Harbin Medical University, Harbin 150081, China

^*^Corresponding authors, Yun-Peng Zhang: [zyp19871208@126.com](mailto:zyp19871208@126.com) and Xia Li: lixia@hrbmu.edu.cn

**Supplementary Information**

**Supplementary Figure S1**. The subpathway number and scale (including gene, miRNA number and miRNA-gene relationships) using different parameters of K (2-4) and T (1-4). A detailed description is included in the Materials and Methods.

**
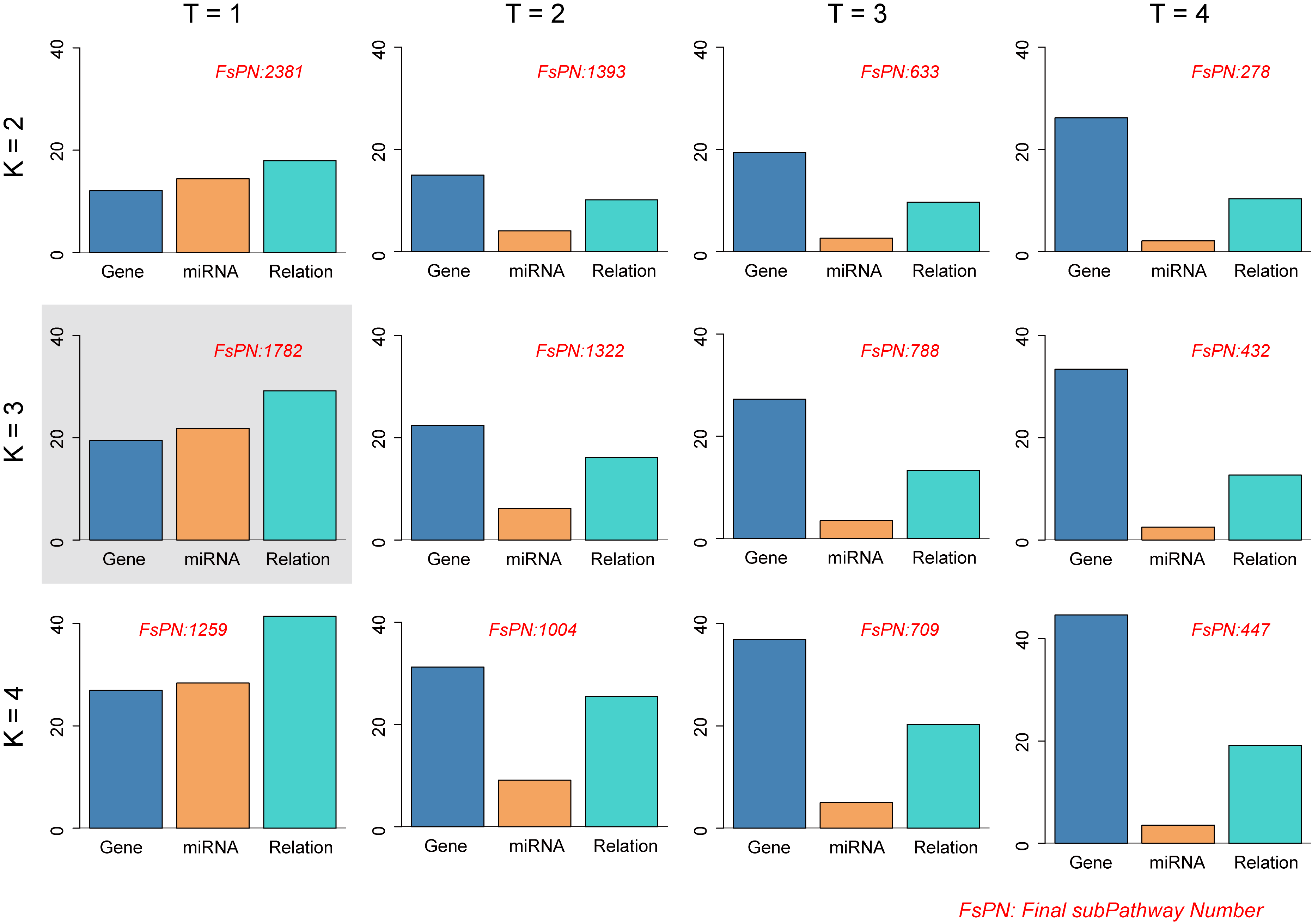
**

**Supplementary Figure S2.** Comparisons between the different reconstructed pathway graphs for (**A**) Small cell lung cancer, (**B**) Non-small cell lung cancer, (**C**) Colorectal cancer, (**D**) Glioma, (**E**) Bladder cancer, (**F**) Prostate cancer and the original pathway graph: MicroRNAs in cancer.

**
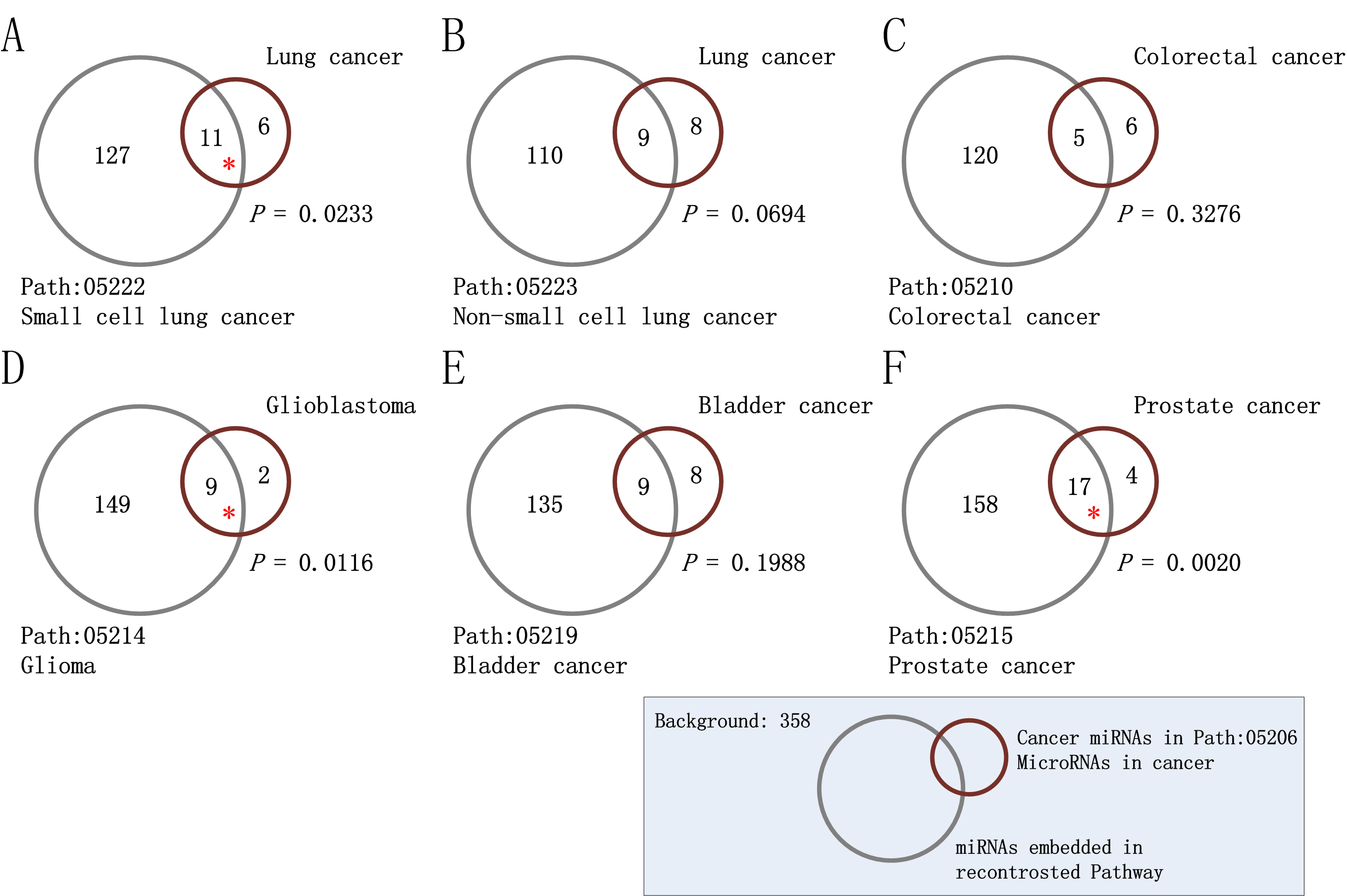
**

**Supplementary Figure S3.** The significances of the clinical outcome differences among four different thyroid cancer clusters were estimated using K-M survival analysis. The P-values were calculated using the log-rank test.

**
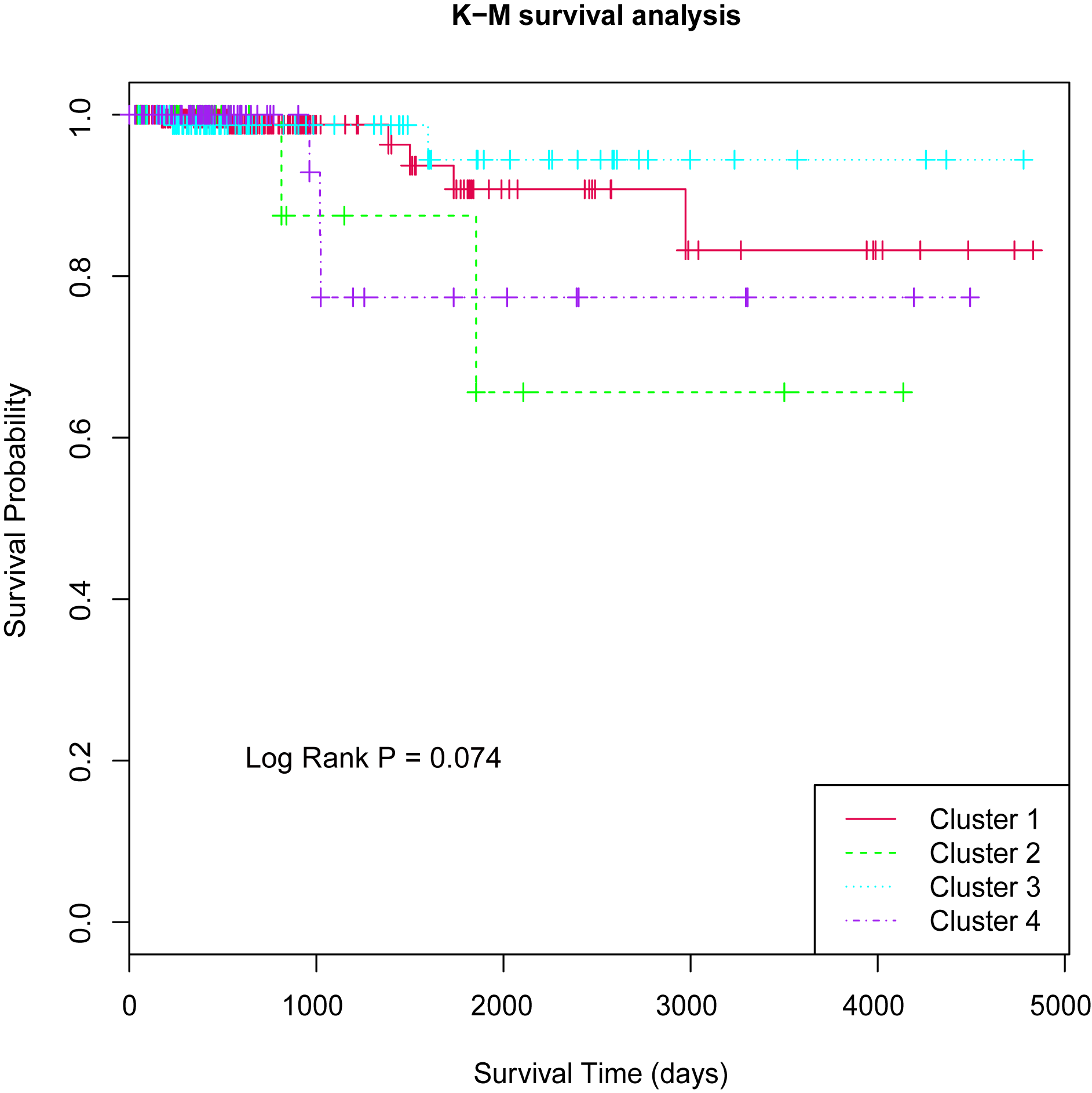
**

**Supplementary Figure S4.** The location and components including embedded miRNAs of two subpathways, Path: 05219_3 and Path: 05219_5. The image was obtained by KEGG^1-3^.

**
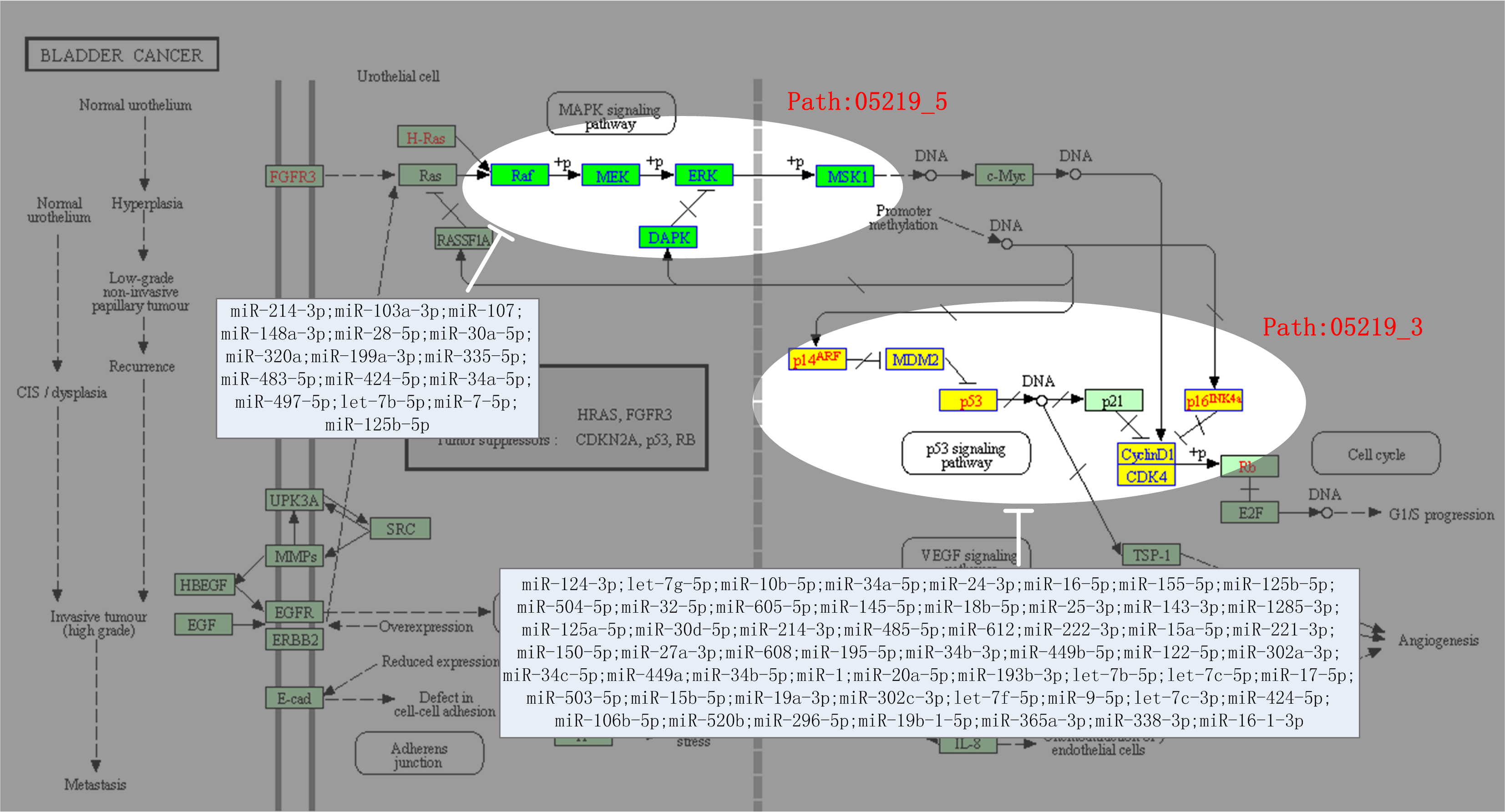
**

**Supplementary Figure S5.** The 55 subpathway signature predicted melanoma patient clinical outcome of TCGA data set. (**A**) K-mean clustering representation of the subpathway signature in the TCGA data set. The columns represented tumor samples and rows represented subpathways. The red color designates high subpathway activity, and the green color designates low activity. (**B**) A K-M plot of the low-risk and high-risk groups. The *P*-value was calculated using the log-rank test.


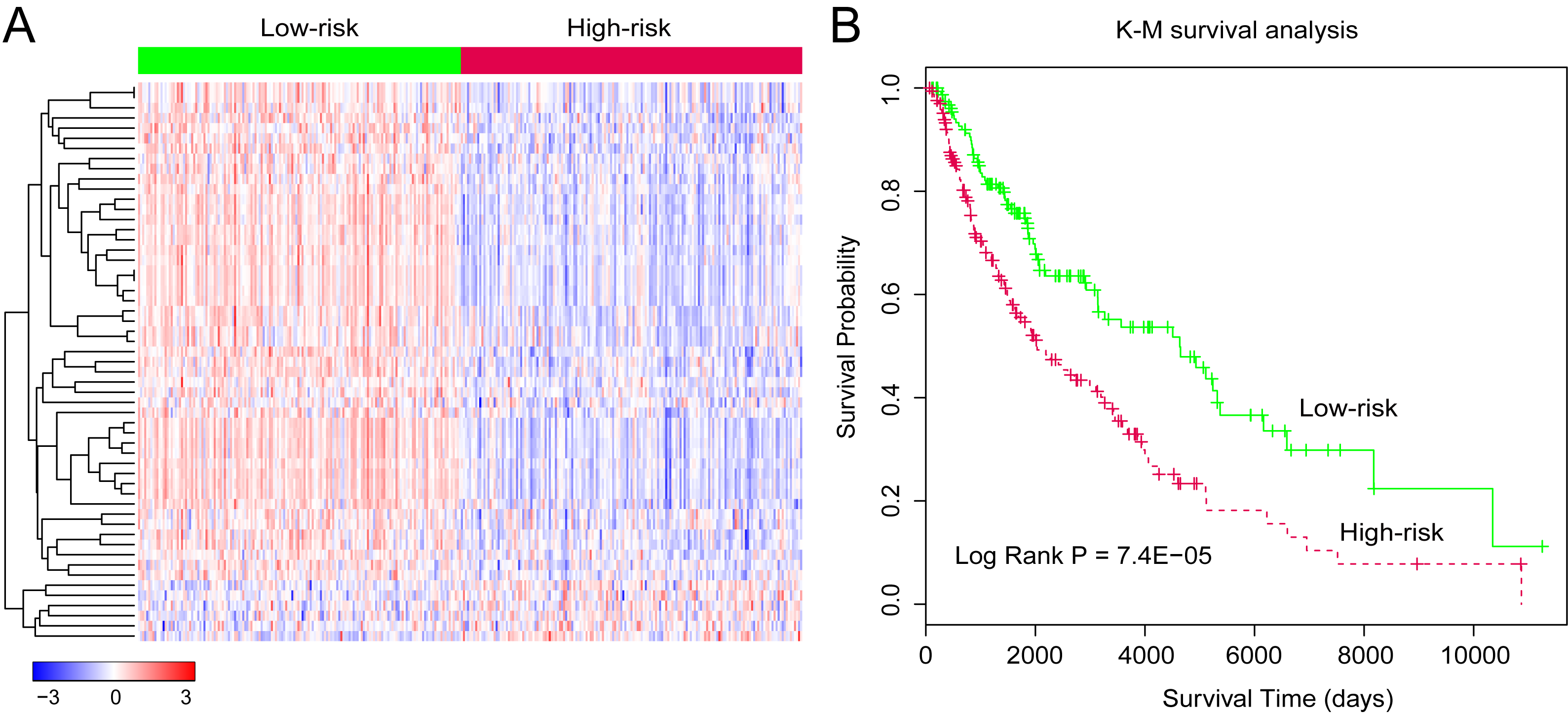


**Supplementary Figure S6.** The Pearson correlations between our model and GSEA (or FAIME) methods for all 4,508 samples of 12 tumor types.


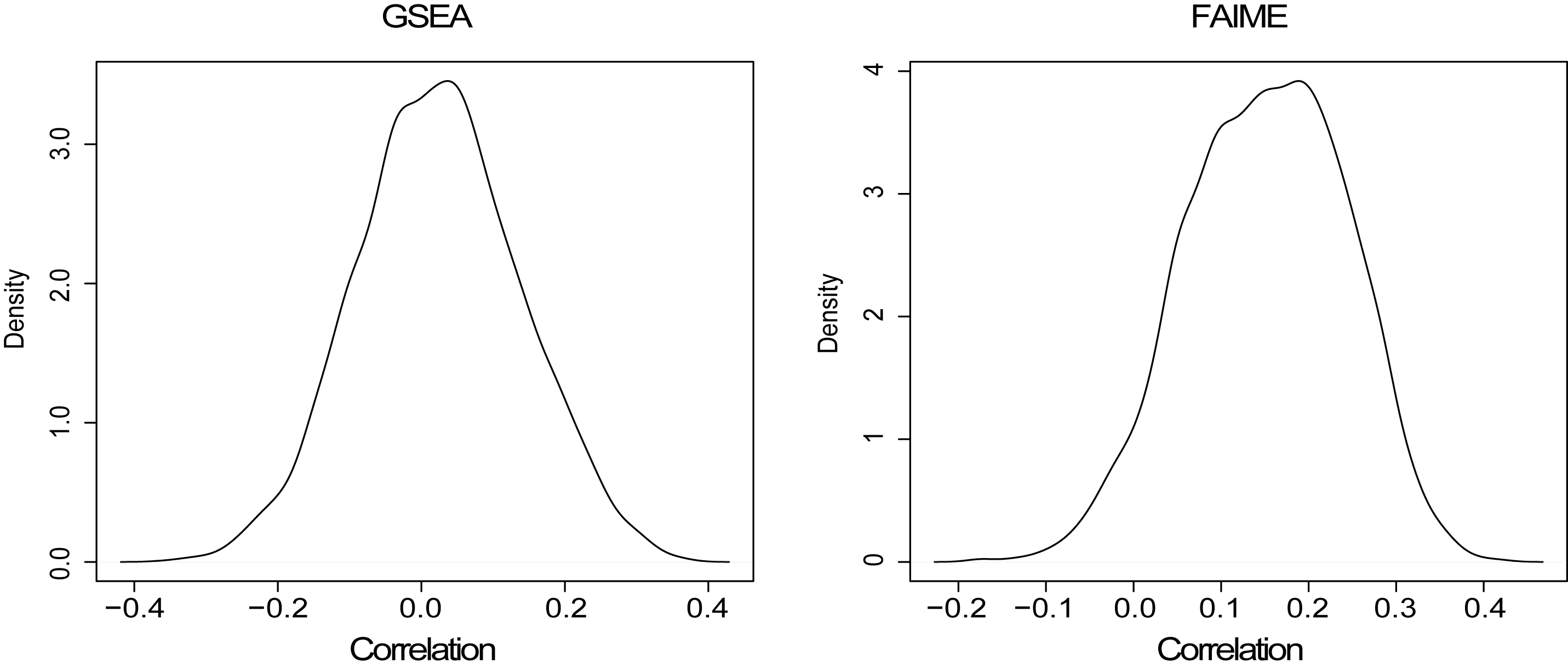


**Supplementary Figure S7.** The Pearson correlations between our model and GSEA (or FAIME) methods for samples of each tumor type.


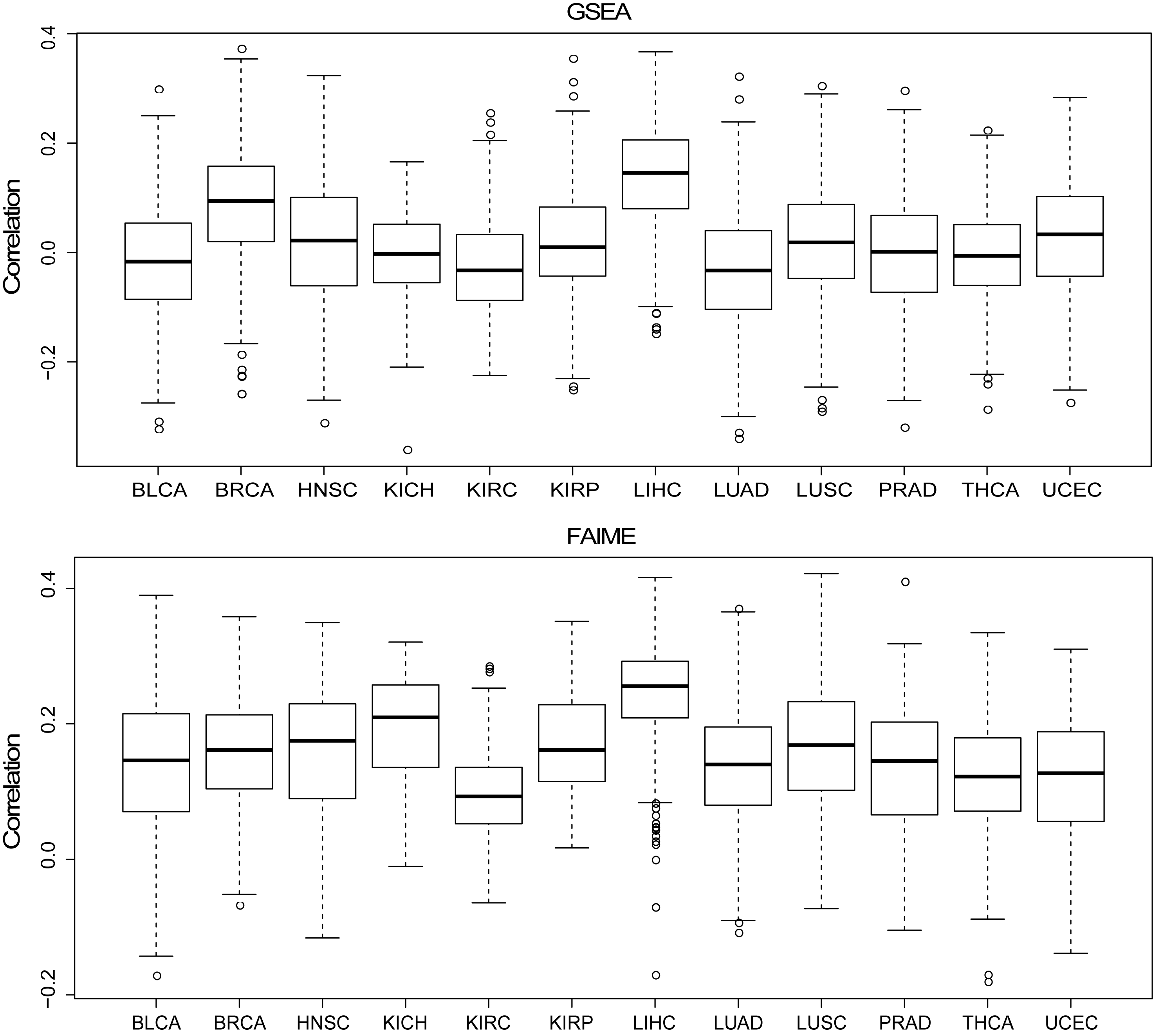


**References**

1 Kanehisa, M., Furumichi, M., Tanabe, M., Sato, Y. & Morishima, K. KEGG: new perspectives on genomes, pathways, diseases and drugs. *Nucleic Acids Res* **45**, D353-D361, doi:10.1093/nar/gkw1092 (2017).

2 Kanehisa, M., Sato, Y., Kawashima, M., Furumichi, M. & Tanabe, M. KEGG as a reference resource for gene and protein annotation. *Nucleic Acids Res* **44**, D457-462, doi:10.1093/nar/gkv1070 (2016).

3 Kanehisa, M. & Goto, S. KEGG: kyoto encyclopedia of genes and genomes. *Nucleic Acids Res* **28**, 27-30, doi:gkd027 (2000).
